# Supplementary material for: Adipocyte-Specific ACKR3 Regulates Lipid Levels in Adipose Tissue
Source: Biomedicines. 2021 Apr 6;9(4):394. doi: 10.3390/biomedicines9040394 (PMC8067615; doi:10.3390/biomedicines9040394)
Supplement: Supplementary file 1 [file biomedicines-09-00394-s001.pdf]

Figure S1. Validation of *Ackr3* deficiency

a

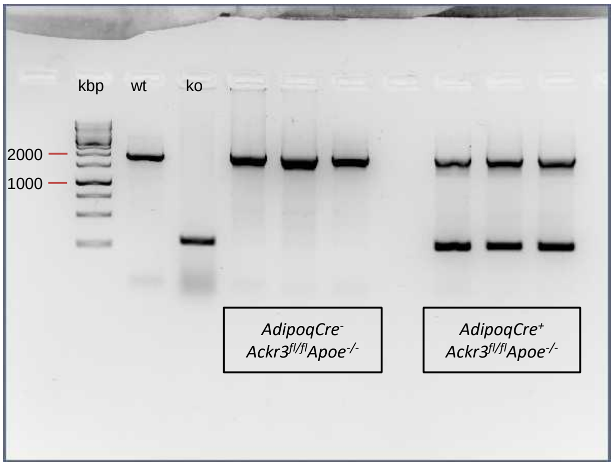

b

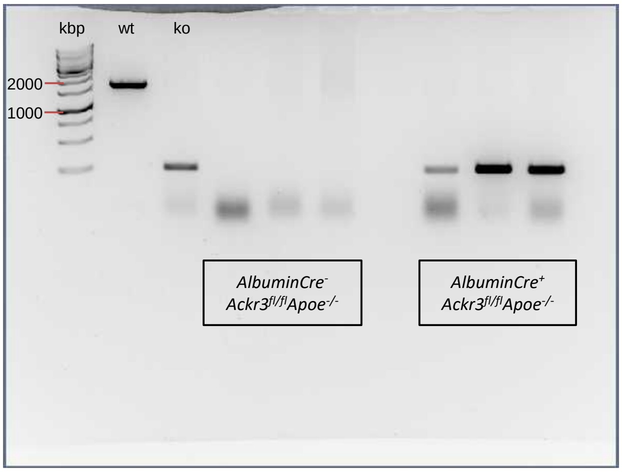

Supplementary Information

Figure S2. Full-length western blot images of Figure 3d.

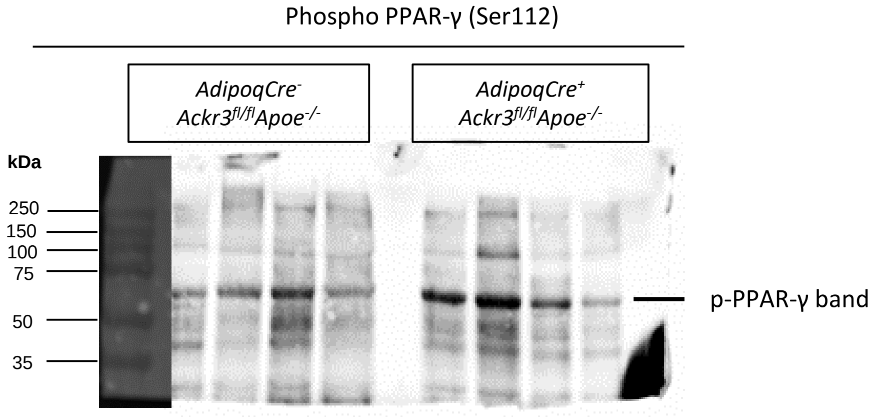

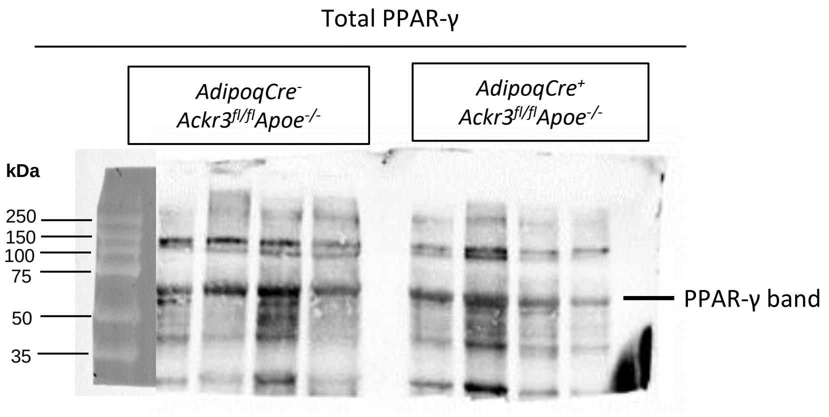

Supplementary Information
